# Systemic sclerosis due to crystalline silica exposure among jewelry workers in Korea: two case reports

**DOI:** 10.1186/s40557-017-0176-x

**Published:** 2017-06-19

**Authors:** Jae Yoon Kim, Sang Yoon Do, Young Hoon Moon, Chul Gab Lee, Yun Sung Kim, Byung Soon Choi, Eun-A Kim, Han Soo Song

**Affiliations:** 1 Department of Occupational and Environmental Medicine, School of Medicine, Chosun University, 558 Pilmun-daero Dong-gu, Gwangju, 61453 Republic of Korea; 2 Division of Rheumatology, Department of Internal Medicine, School of Medicine, Chosun University, Gwangju, Republic of Korea; 3 Occupational Lung Diseases Institute, Korea Workers’ Compensation and Welfare Service, Incheon, Republic of Korea; 4 Occupational Safety and Health Research Institute, Korea Occupational Safety & Health Agency, Ulsan, Republic of Korea

**Keywords:** Crystalline silica, Jewelry, Systemic sclerosis, Occupational exposure, Korea

## Abstract

**Background:**

Occupational exposure to crystalline silica is a potential risk factor for various systemic autoimmune diseases including systemic sclerosis. The etiology of systemic sclerosis is not conclusively known, but there are epidemiological studies that show the relationship between exposure to crystalline silica and risk of systemic sclerosis. Here we report, for the first time, two cases of crystalline silica-related systemic sclerosis in patients who worked in crystal processing in the jewelry-manufacturing field.

**Case presentation:**

Case 1 is a 57-year-old man who had worked mainly in crystal processing for multiple jewelry-processing companies for 17 years, since the age of 15 years. He contracted tuberculosis at the age of 25 years and showed Raynaud’s phenomenon of both the hands and feet at age 32 years. Digital cyanosis and sclerosis developed at approximately age 41 years. The patient was diagnosed with systemic sclerosis at age 48 years.

Case 2 is a 52-year-old man who worked in crystal processing for various jewelry-processing companies for 7 years, since the age of 23 years. He first showed signs of cyanosis in the third and fourth digits of both hands at age 32 years, was diagnosed with Raynaud’s syndrome at age 37 years, and was diagnosed with systemic sclerosis at age 38 years.

Crystal processing is a detailed process that involves slabbing and trimming the selected amethyst and quartz crystals, which requires close proximity of the worker’s face with the target area. In the 1980s and 1990s, the working hours were 12 h per day, and the working environment involved 15 workers crowded into a small, 70-m^2^ space with poor ventilation.

**Conclusion:**

Two workers who processed crystals with a maximum crystalline silica content of 56.66% developed systemic sclerosis. Considering the epidemiological and experimental evidence, exposure to crystalline silica dust was an important risk factor for systemic sclerosis. An active intervention is necessary to reduce exposure in similar exposure groups in the field of jewelry processing.

## Background

Silica exists in crystalline and non-crystalline forms. The most common natural form of crystalline silica is quartz. Quartz is a colorless, odorless, non-combustible solid that widely exists in rocks, sand, and soil. Quartz causes silicosis via occupational respiratory exposure to the mineral dust form [1]. Miners, sandblasters, foundry workers, tunnel drillers, quarry workers, stone carvers, ceramic workers, and silica flour production workers are at a potential risk of exposure to crystalline silica [2]. When silicosis occurs simultaneously with scleroderma, it is called Erasmus syndrome. When silicosis and rheumatoid arthritis coexist, it is called Caplan’s syndrome. As such, research shows that exposure to crystalline silica is not limited to the lungs but is also related to systemic autoimmune disease [3]. A recent review on environmental factors that affect autoimmune disease reported epidemiological evidence that exposure to crystalline silica contributes to the occurrence of diseases such as rheumatoid arthritis, systemic sclerosis, systemic lupus erythematosus, and antineutrophil cytoplasmic antibody-related vasculitis [4].

Systemic sclerosis is a connective tissue disease characterized by microvascular occlusive disease, small artery proliferative disease, and the accumulation of collagen and extracellular matrix components in the skin and interstitium of target internal organs [5]. Systemic sclerosis is a rare disease with a widely varying incidence between 0.6 and 233 persons per million per year, depending on the region. The incidence in women is reported to be up to 14 times higher than that in men [6]. The etiology of systemic sclerosis remains unclear, but environmental factors are thought to play an important role in addition to genetic and autoimmune factors [7]. The possible occupational risk factors such as crystalline silica, mineral spirits, aromatic solvents, chlorinated solvents, trichloroethylene, ketones, and welding fumes have been reported to be associated with systemic sclerosis [8].

In Korea, there were two approved cases of industrial accident compensation for systemic sclerosis. The first case was approved in 1999 and was observed in a female worker who did bonding work in a tennis ball production company for 15 years. She was thought to have been exposed to high concentrations of aromatic hydrocarbons, with toluene being the main component. She first developed edema of the hands, then skin keratinization symptoms. This was accompanied by interstitial pneumonia, axonal peripheral neuropathy, cortical atrophy, multiple cerebral infarction, and memory impairment [9]. The second case was approved in 2002 and was observed in a male worker who engaged in crushing aggregates for 21 years. He was first diagnosed with tuberculosis 6 years after the cessation of exposure, and then was diagnosed with pneumoconiosis and scleroderma [10].

We have confirmed that the two workers who visited our occupational & environmental medicine clinic for industrial accident compensation for Raynaud’s syndrome suffer from secondary Raynaud’s syndrome due to systemic sclerosis rather than isolated Raynaud’s syndrome. An enquiry into occupational history revealed that the patients worked in crystal processing at the Iksan Precious Metal Complex. Crystal processing involves measuring, cutting, grinding, and polishing the raw crystal. Many workers were crowded into a small space. Crystals such as amethyst and quartz, unlike cubic zirconia, jade, ruby, or sapphire, have a high crystalline silica content in the raw ore and would have exposed the workers to high levels of crystalline silica.

Occupations that have previously been connected to crystalline silica-related systemic sclerosis include gold, coal, or iron miners, stone quarry cutters, mining shotfirers, ceramics workers, millers, stonemasons, and stone crushers [11–21]. However, there has never been a reported case of crystalline silica-related systemic sclerosis in crystal processing workers in the jewelry-processing field. Hence, we report two cases of systemic sclerosis in crystal processing workers in the jewelry-processing field who are thought to have been exposed to crystalline silica dust; we also present a literature review.

## Case presentation

### Case presentation 1

#### Patient information

Fifty-seven-year-old man

#### Chief complaint

**Fig. 1:**
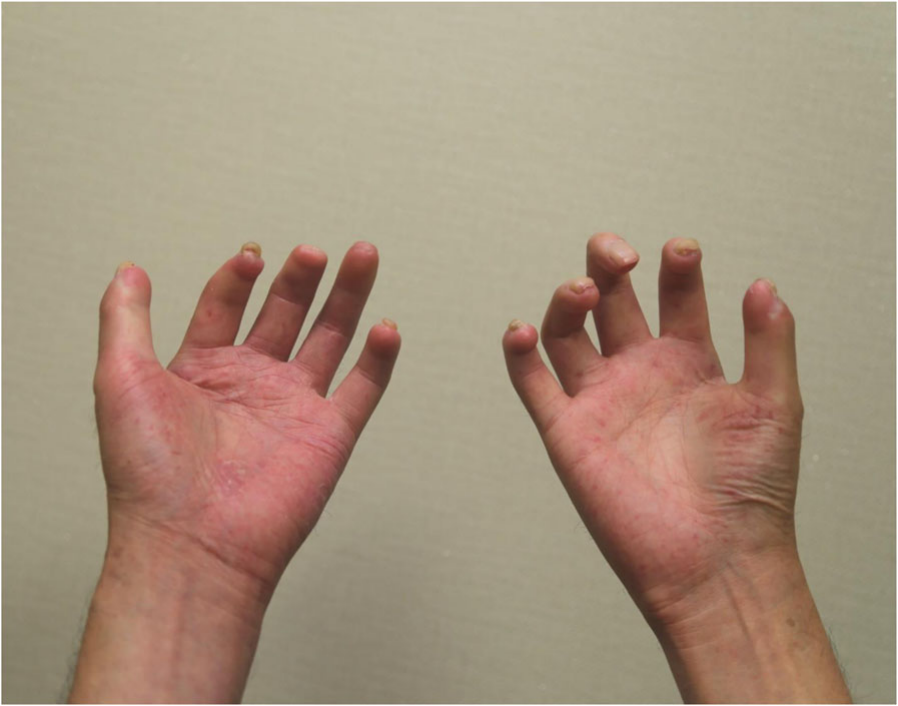
Hands of Case 1. All the fingers of this patient’s hands show joint contractures, and the distal phalanx of the left third finger was self-amputated

Digital necrosis and contracture associated with swelling in cold weather, cyanosis, and tingling (Fig. 1).

#### Past medical history

The patient worked in crystal processing in the jewelry-processing industry for 17 years from the age of 17 years in 1976 to January 1994. In 1984, he was first diagnosed with pulmonary tuberculosis, which recurred but was cured in 2000. He first experienced Raynaud’s phenomenon of both the hands and feet in 1991. He had a chest tube inserted in 1993 due to left pneumothorax. In approximately the year 2000, he developed digital cyanosis and hardening. In April 2006, he reported swelling, pain, and gangrenous lesions of the right great toe and left third finger. He subsequently presented to a local university hospital, where he was first diagnosed with systemic sclerosis. His symptoms progressed to digital contracture and auto-amputation of the distal phalanx of the left third finger due to necrosis. He suffered from a generalized tonic-clonic seizure (GTCS) in September 2006.

#### Smoking history

Non-smoker

#### Alcohol history

Non-drinker

#### Occupational history

The patient worked in crystal processing for the Iksan Precious Metal Complex for approximately 17 years, from 1976 to 1994. He stopped working at approximately age 35 years when he first developed symptoms and has not worked since.

#### Physical examination

Upon presentation in August 2015, the patient was found to have amputation of the distal phalanx of the left third finger and limited movement of the first and second phalanges due to sclerodactyly. He was also found to have telangiectasia of the hands and face.

#### Laboratory findings

According to the results of the laboratory tests conducted on September 1, 2015 in rheumatology department of our hospital, the white blood cell (WBC) count, red blood cell (RBC) count, and platelet count were within normal limits. Thyroid function tests were within normal limits, with free thyroxine (T4), 1.76 ng/dL; triiodothyronine (T3), 81.5 ng/dL; and thyroid-stimulating hormone (TSH), 1.14 μIU/mL. The erythrocyte sedimentation rate (ESR) was elevated to 52 mm/h (normal <20 mm/h), and C-reactive protein (CRP) was also elevated, at 3.09 mg/dL (normal <0.3 mg/dL). Anti-Scl (scleroderma)-70 antibody was positive at 106.8 U/mL (reference range < 15 U/mL). Fluorescent antinuclear antibody (FANA) was positive with a fluorescent intensity of 4+, a homogenous type and a titer of 1:640. Other autoimmune markers such as rheumatoid factor, anti-CCP antibody (anti-cyclic citrullinated peptide antibody), and anti-centromere antibody were negative. According to the medical records brought by the patient, FANA (homogenous + nucleolar type) and anti-Scl-70 were positive in 2006.

#### Radiological findings

**Fig. 2:**
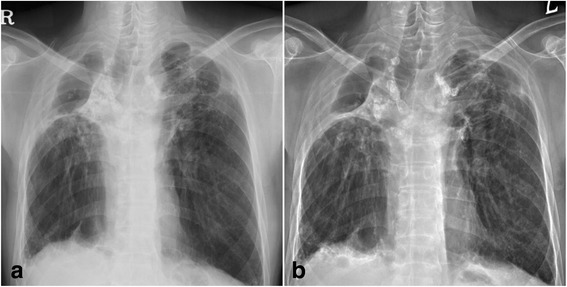
Simple chest radiographs of Case 1. **a** In 2011, pulmonary tuberculosis sequelae with fibrosis scarring were observed. Reticulations were observed in both lower lobes. Pleural thickening and calcification were found in the right lower lung. **b** In 2016, fibrocalcifications of both the upper lungs and the right lower lung were observed. Emphysematous bullae were observed in both the upper lungs. The reticular pattern and number of bullae increased as compared with 2011

In the simple chest radiograph taken at a different center in 2011, there was evidence of pulmonary tuberculosis sequelae, with observed bilateral lung volume loss, fibrosis scarring and distortion, and bronchiectasis with cicatrization. The patient was diagnosed with combined pulmonary fibrosis with emphysema(CPFE) after observing a reticular pattern in both the lower lungs and pleural thickening and calcification of the right lower lung. In the simple chest radiograph taken at our hospital in 2016, we observed emphysematous bullae in both the upper lungs, associated with cicatricial changes as well as fibrocalcification of both the upper lungs and the right lower lung. The reticular pattern and number of bullae increased since 2011, showing an overall deterioration (Fig. 2).

#### Other tests

Echocardiography performed in our hospital showed mild pulmonary hypertension with a pulmonary artery systolic pressure (PASP) of 51 mmHg. Nailfold capillary microscopy showed capillary atrophy, which suggests chronic-stage systemic sclerosis. Hand perfusion scintigraphy showed mildly decreased perfusion and blood pooling of the chilled finger to about 81% compared to the opposite finger.

#### Diagnostic assessment

The patient showed classic sclerodactyly of the hands, digital tip ulcers and pitting scars, and telangiectasia. He also showed abnormal nailfold capillaries as well as mild pulmonary hypertension by echocardiography. He was diagnosed with interstitial lung disease as per radiological evidence, and he had a past medical history consistent with Raynaud’s syndrome. He also showed decreased perfusion by hand perfusion scintigraphy. He was anti-Scl-70 antibody positive. Based on the above findings, he fulfilled one major criterion (proximal scleroderma) and three minor criteria (sclerodactyly, digital pitting scars of the fingertips or loss of substance of the distal finger pad, bilateral basilar pulmonary fibrosis) according to the American Rheumatism Association (ARA) criteria (1980) [22]. Based on scoring using the American College of Rheumatology/European League Against Rheumatism Collaborative Initiative (ACR-EULAR) Classification Criteria for Systemic Sclerosis (2013) [23], the patient also fulfills the diagnostic criteria, thus confirming the diagnosis of definite systemic sclerosis (Table 1).

**Table 1:** Diagnostic criteria for systemic sclerosis (American College of Rheumatology/European League Against Rheumatism criteria for the classification of systemic sclerosis [23])

Item	Sub-item(s)	Weight/score ^a^	Case 1	Case 2
Skin thickening of the fingers of both hands extending proximal to the metacarpophalangeal joints (sufficient criterion)		9	●	●
Skin thickening of the fingers (only count the higher score)	Puffy fingers	2	●	●
	Sclerodactyly of the fingers (distal to the metacarpophalangeal joints but proximal to the proximal interphalangeal joints)	4	●	●
Fingertip lesions (only count the higher score)	Digital tip ulcers	2	●	●
	Fingertip pitting scars	3	●	●
Telangiectasia		2	●	●
Abnormal nailfold capillaries		2	●	○
Pulmonary arterial hypertension and/or interstitial lung disease (maximum score is 2)	Pulmonary arterial hypertension	2	●	●
	Interstitial lung disease	2	●	●
Raynaud’s syndrome		3	●	●
SSc-related autoantibodies (anti-centromere, anti– topoisomerase I [anti–Scl-70], anti–RNA polymerase III) (maximum score is 3)	Anti-centromere	3	N	N
	Anti–topoisomerase I		●	●
	Anti–RNA polymerase III		○	○

^a^The total score is determined by adding the maximum weight (score) in each category. Patients with a total score of nine or higher are classified as having definitive scleroderma

● yes; ○ no confirmation; N negative

#### Therapeutic interventions

He is currently undergoing medical treatment in rheumatology department of our hospital every 4 weeks. The drugs he is taking are prednisolone, platelet aggregation inhibitor, angiotensin II receptor antagonist, nonsteroidal anti-inflammatory drug, xanthine oxidase inhibitor and endothelin receptor antagonist.

#### Follow up and outcomes

He was approved for industrial accident compensation from Korea Workers’ Compensation & Welfare Service (KCOMWEL) for systemic sclerosis in 2016. His symptoms are no longer worse and remain similar. In winter, however, deterioration of the hardness of his fingers often occurs.

### Case presentation 2

#### Patient information

Fifty-two-year-old man

#### Chief complaint

Gradual stiffening of the fingers

#### Past medical history

The patient worked in crystal processing in the jewelry-processing field for 7 years from the age of 23 years, between 1987 and 1993. He first developed cyanosis of the third and fourth fingers up to the third phalanx of both the hands in the winter of 1996. He was admitted to a university hospital in the winter of 2001 for a severe sensation of cold in the fingers, and he was diagnosed with Raynaud’s syndrome. He was diagnosed with systemic sclerosis in a tertiary hospital in Seoul after presenting with a chapping digital tip ulcer. Subsequently, he developed hardening of the fingers and dyspnea on exertion. He was diagnosed with pulmonary artery hypertension in 2009 and with interstitial pneumonia in 2014. After leaving the jewelry-processing company and starting work as a forklift technician, his symptoms worsened and improved repeatedly.

#### Smoking history

The patient started smoking during his military service and had repeated periods of smoking and non-smoking. Currently, he rarely smokes, and on these occasions only smokes one or two cigarettes. He has a 30 pack-year history in total.

#### Alcohol history

Non-drinker

#### Occupational history

He served 3 years in the army after graduating high school. He subsequently worked as a crystal processor in a company within the Iksan Precious Metal Complex for 7 years between 1987 and 1993. After this, he worked in forklift repair for 12 years, then as a wildfire monitor for 5 years.

#### Physical examination

Upon presentation to our clinic in September 2015, the patient had contractures of the finger joints as well as trophic change of the fingers. He also had bilateral auto-amputation of the tips of the second and third fingers.

#### Laboratory findings

Laboratory findings on October 13, 2015 at rheumatology department of our hospital showed RBC, WBC, and platelet counts within normal limits, as well as normal thyroid function tests with free T4, 1.11 ng/dL; T3, 135.6, ng/dL; and TSH, 2.14 μIU/mL. ESR was elevated to 40 mm/h (normal <20 mm/h), and CRP was also raised, to 0.54 mg/dL (normal <0.3 mg/dL). The patient was antinuclear antibody positive, with a fluorescent intensity of 4+, a homogenous pattern, and a titer of 1:1280. It was concluded that the pattern was similar to that of anti-Scl-70. Other autoimmune markers such as rheumatoid factor and anti-centromere antibody were negative.

#### Radiological findings

**Fig. 3:**
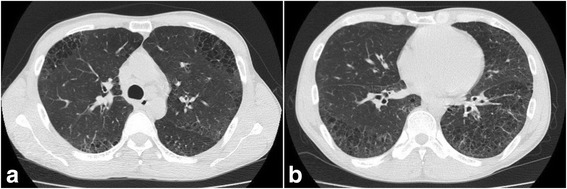
Chest computed tomography image of Case 2. **a** In both the upper lungs, cystic lesions with bronchiectasis were observed. **b** Peripheral reticulations and cystic lesions were found in both lower lobes

According to chest computed tomography performed in a tertiary hospital in Seoul on June 26, 2014, the patient showed peripheral predominant reticulation and cystic lesions in both lungs, consistent with systemic sclerosis-associated interstitial lung disease (Fig. 3).

#### Other tests

Echocardiography performed in April 2015 at a tertiary hospital in Seoul showed borderline pulmonary artery hypertension (PASP 37 mmHg). Hand perfusion scintigraphy performed by the nuclear medicine department in our hospital showed a marked decrease in perfusion and blood pooling of the chilled finger to 22% of the ambient hand finger.

#### Diagnostic assessment

The patient showed classic sclerodactyly of both the hands, digital tip ulcers and depressed scars, and telangiectasia. He showed borderline pulmonary hypertension on echocardiography and interstitial lung disease on chest computed tomography in 2014. He also showed symptoms and signs of Raynaud’s syndrome as well as markedly decreased perfusion on hand perfusion scintigraphy, consistent with Raynaud’s syndrome. Based on the above findings, the patient fulfilled one major and three minor ARA criteria (1980) [22] as well as diagnostic criteria based on ACR-EULAR Classification Criteria for Systemic Sclerosis (2013) [23] scoring, thus confirming the diagnosis of definitive systemic sclerosis (Table 1).

#### Therapeutic interventions

He is currently undergoing medical treatment in rheumatology department of our hospital every 3 months. The drugs he is taking are calcium channel blocker, anti-inflammatory analgesic drug, endothelin receptor antagonist and mucolytics.

#### Follow up and outcomes

He received industrial accident compensation for systemic sclerosis in 2016 from KCOMWEL. His symptoms are no longer worse and remain similar. In winter, however, digital tip ulcers often occur.

## Discussion

### Review of epidemiologic studies

In 1957, Erasmus reported 17 cases of systemic sclerosis in miners who worked in the mines of South Africa [24]. This report led to the naming of crystalline silica exposure-related systemic sclerosis as Erasmus syndrome. There have since been various reports of systemic sclerosis due to exposure to crystalline silica. McCormic et al. conducted a meta-analysis of 16 studies published between 1949 and November 2009 that report the relationship between crystalline silica exposure or silicosis and systemic sclerosis. This meta-analysis shows that, based on 9 case–control studies, the combined estimator of relative risk (CERR) of patients who have been exposed to crystalline silica compared to those without crystalline silica exposure was 2.24 (95% confidence interval (CI), 1.65–3.31). The CERR based on three large-scale cohort studies conducted in Sweden, Denmark, Germany, and the USA was 15.49 (95% CI, 4.54–52.87) [7].

An in vitro study by Haustein et al. showed that crystalline silica can activate microvascular endothelial cells, peripheral blood mononuclear cells, and skin fibroblasts in a manner similar to the pathophysiologic phenomenon of idiopathic systemic sclerosis [25]. A recent, related study reported that crystalline silica triggers a pre-fibrotic reaction in fibroblasts by stimulating gene expression in the extracellular matrix [26]. In a rat model of crystalline silica exposure, an autoimmune reaction related to scleroderma was identified [27], providing evidence for the relationship between silica exposure and autoimmunity in animal models.

However, there is no study to date on the relationship between crystalline silica exposure and systemic sclerosis. In a cohort study of 5414 male stone workers who worked in the state of Vermont between 1924 and 1977, the cumulative exposure to respirable crystalline silica of 0.5 mg-yr./m^3^ or more resulted in a standardized mortality rate (SMR) from malignant tumors of the airway, bronchus, and lungs that is 1.42 times higher (SRR; standardized ratio rate 2.14, *p* < 0.05), and a cumulative exposure of 2.0 mg-yr./m^3^ or more resulted in an SMR that is 1.88 times higher (SRR 2.60, *p* < 0.01) [28]. There was no data regarding a quantitative assessment of the level of exposure while working for the jewelry-processing company.

### The possibility of exposure to crystalline silica, and the exposure level

The last company for which Case 1 worked, for 1 month before he retired from the jewelry-processing industry, and the last company for which Case 2 worked, for 5 years, are the same company in the Precious Metal Complex. It was the biggest company in the complex, with approximately 500 employees. It is impossible to check the work environment, because the company has now moved to China. However, according to the statement given by Case 2, there were over 20 processing rooms that were approximately 70 m^2^ in area in a two-story building, with approximately 15 workers in one room dividing the work. There were a window and two small ventilators on one of the walls, but it was located high up on the wall. The window was a sliding window with a small height and large width, which was stated to be a measure to prevent the workers from stealing the jewels. The patients stated that the ventilation within the room was inadequate due to the aforementioned structure and caused the room to be constantly dusty.

The work mainly involved measuring and cutting the selected raw crystals for appropriate use, which included slabbing and trimming. This process requires the worker to cut the crystal with a chain saw. The worker would have to bring his face close to the area of work in order to make precise cuts in small crystals. Case 1 stated that the coolant and dust came in direct contact with his face as the chainsaw was operating. The cutting, grinding, and polishing of the crystals occurred constantly, and the dust from this process accumulated in the processing room. Case 1 also stated that the cooling water was repeatedly re-used without being replaced. The working hours at the time were from 7 am to 9 pm, totaling approximately 12 h per day excluding meals and breaks, and the workers were given 1 day off every 2 weeks.

### Exposure assessment

There is very limited data on the level of exposure to crystalline silica in those working in the jewelry processing business. According to the work-related assessment conducted in December 1999 by the Occupational Safety & Health Research Institute, Korea Occupational Safety & Health Agency (Tables 2 and 3), the content of crystalline silica in amethyst, one of the materials handled at jewelry-processing companies in the Iksan Precious Metal Complex, was 8.25–42.43%, and the content of crystalline silica in quartz was 56.66%. The level of exposure to crystalline silica in the jewelry-processing business for the Iksan Precious Metal Complex was 0.025 mg/m^3^(not detected to 0.082 mg/m^3^). The “not detected” result is derived from the fact that the processing work included a significant amount of cubic zirconia, ruby, and sapphires, which do not contain crystalline silica. The maximum exposure to crystalline silica was 0.082 mg/m^3^, which exceeds the time-weighted average standard of 0.05 mg/m^3^ for crystalline silica, as specified by the Ministry of Employment and Labor. The duration of exposure was 17 years in Case 1 and 7 years in Case 2. Both patients had closer exposure to the respiratory tract than normal for 12 h a day. There was a case of International Labour Organization classification of pneumoconiosis with category 2/2 profusion in a jewelry-processing worker who worked in the industry for 27 years.

**Table 2:** Content of crystalline silica in raw materials – the Iksan Precious Metal Complex Jewelry Processing Company (1999)

Company	Raw material	Crystalline Silica Content (%)
Company A	Cubic zirconia	ND
Company B	Jade	ND
Company C		
First Partner	Amethyst	15.23%
Second Partner	Cubic zirconia	ND
Third Partner	Amethyst	42.43%
Company D	Amethyst	8.25%
Company E	Cubic zirconia	ND
Company F	Cubic zirconia, Ruby, Sapphire	ND
Company G	Cubic zirconia	ND
Company H	White crystal	56.66%

*ND* not detected

**Table 3:** Exposure level to dust by raw materials – the Iksan Precious Metal Complex Jewelry Processing Company (1999)

	Crystal		Non-Crystal	
	No. of Personal Samples	Exposure Level (mg/m^3^)	No. of Personal Samples	Exposure Level (mg/m^3^)
Total Dust	11	0.642 (0.267–1.841)	38	0.254 (0.045–0.675)
Respirable dust	9	0.219 (0.101–0.388)	18	0.108 (0.010–0.281)
Crystalline silica	9	0.025 (ND^a^ - 0.082)	18	ND

*ND* not detected

^a^The two workers mainly processed cubic zirconia, ruby, sapphires, and sometimes amethyst

### Clinical features

Freire et al. reported the clinical features of systemic sclerosis due to crystalline silica exposure based on case-cohort studies conducted by the authors themselves as well as prior case report studies. According to this report, Raynaud’s syndrome occurred in 81% of patients with crystalline silica exposure-related systemic sclerosis, digital ulcers in 48%, interstitial lung disease in 78%, silicosis in 40%, and anti-Scl-70 positivity in 44%. In addition, patients who were exposed to crystalline silica developed the above conditions early and had a worse prognosis compared with controls, who were not exposed to crystalline silica [29].

The two cases reported herein worked in crystal processing in the jewelry-processing field. They first developed Raynaud’s phenomenon of both the hands, and then developed digital cyanosis and hardening. They subsequently developed digital tip ulcers that chapped, progressing to digital contractures, fingertip necrosis, and auto-amputation. They were subsequently diagnosed with pulmonary artery hypertension and interstitial lung disease. Both patients were diagnosed with definitive systemic sclerosis, having fulfilled the ACR-EULAR Classification Criteria for Systemic Sclerosis (2013) [23]. An interesting fact to note was the generalized tonic-clonic seizure that developed 5 months after the diagnosis of systemic sclerosis in Case 1. The T2-weighted brain magnetic resonance image showed a high signal intensity in the pons, as well as multifocal high signal intensity in the bilateral basal ganglia, bilateral frontal and occipital cortices, and subcortical white matter. There were no abnormal radiologic or echocardiographic findings, nor were there blood count or electrolyte abnormalities, allowing us to rule out underlying pathology other than systemic sclerosis that might precipitate a GTCS. Seizures in patients with systemic sclerosis are very rare worldwide. In Korea, the first case of seizure in a patient with systemic sclerosis was reported in 2004 by Im et al. [30].

## Conclusion

We confirmed two relatively rare cases of systemic sclerosis in crystal processing workers in the jewelry-processing field who worked in the Iksan Precious Metal Complex. Crystalline silica is considered a possible environmental risk factor for systemic sclerosis based on relatively clear evidence based on epidemiologic and animal studies. Considering the occupational history of the two patients, it can be assumed that they were exposed to a considerable level of crystalline silica while processing crystals with abundant crystalline silica contents in a poorly ventilated location.

We concluded that the two cases were strongly related to occupation, and Korea Workers’ Compensation & Welfare Service (KCOMWEL) approved industrial accident compensation in 2016. These case reports are meaningful, as they report on systemic sclerosis in the field of jewelry processing, which was not previously considered an occupation with high-level exposure to crystalline silica. Because there is a possibility that those working in similar fields will develop autoimmune disease such as systemic sclerosis, active measures are necessary to take caution and reduce exposure at work.

## Abbreviations

ACR-EULAR: American College of Rheumatology/European League Against Rheumatism Collaborative Initiative
Anti-CCP Ab: Anti-cyclic citrullinated peptide antibody
ARA: American Rheumatism Association
CERR: Combined estimator of relative risk
CPFE: Combined pulmonary fibrosis with emphysema
CRP: C-reactive protein
ESR: Erythrocyte sedimentation rate
FANA: Fluorescent antinuclear antibody
GTCS: Generalized tonic-clonic seizure
KCOMWEL: Korea Workers’ Compensation & Welfare Service
PASP: Pulmonary artery systolic pressure
RBC: Red blood cell
SMR: Standardized mortality rate
SRR: Standardized ratio rate
TSH: Thyroid-stimulating hormone
WBC: White blood cell
